# Depression and Anxiety Among Patients with Epilepsy: A Cross-Sectional Study in Saudi Arabia

**DOI:** 10.3390/brainsci15050484

**Published:** 2025-05-05

**Authors:** Mohammed A. Aljaffer, Ahmad H. Almadani, Ayedh H. Alghamdi, Anas A. Alalwan, Fay H. Albuqami, Khalid O. Altowaijri, Lujain A. Alkhalaf, Faisal A. Alazmi, Shahad F. Aljeri, Hadel H. Alhaluli, Bandar N. Aljafen

**Affiliations:** 1 Department of Psychiatry, College of Medicine, King Saud University, Riyadh 11451, Saudi Arabia; maljaffer@ksu.edu.sa (M.A.A.); aayedh@ksu.edu.sa (A.H.A.); 2 Department of Psychiatry, King Saud University Medical City, King Saud University, Riyadh 11362, Saudi Arabia; ianas.alalwan@gmail.com (A.A.A.); albuqamifay@gmail.com (F.H.A.); 3 SABIC Psychological Health Research and Applications Chair (SPHRAC), Department of Psychiatry, College of Medicine, King Saud University, Riyadh 12372, Saudi Arabia; 4 College of Medicine, King Saud University, Riyadh 11451, Saudi Arabia; khalidaltowaijeri@gmail.com (K.O.A.); lujainahmed441m@gmail.com (L.A.A.); fsal045@gmail.com (F.A.A.); shahadaljeri@gmail.com (S.F.A.); hadeelalhaluli4@gmail.com (H.H.A.); 5 Neurology Unit, Department of Medicine, College of Medicine, King Saud University, Riyadh 11451, Saudi Arabia; baljafen@ksu.edu.sa

**Keywords:** anxiety, depression, epilepsy, quality of life, Saudi Arabia

## Abstract

**Background:** Epilepsy is a major neuropsychiatric disorder affecting many people worldwide, with depression and anxiety being common comorbidities that impact the quality of life (QoL). This study aims to examine depression and anxiety in epileptic patients at a tertiary care hospital, King Khalid University Hospital (KKUH), Riyadh, Saudi Arabia. It also aims to assess participants’ QoL and explore associated risk factors. **Methods:** This cross-sectional study enrolled 400 participants using a convenience sampling technique. The study tool consisted of a questionnaire, the Arabic version of the Hospital Anxiety and Depression Scale, and the Arabic version of the Quality of Life in Epilepsy Inventory. **Results:** The results revealed that 48.25% of the participants exhibited depression, and 39.75% exhibited anxiety. There was a statistically significant association between depression and educational level, employment status, history of psychiatric disorders, epilepsy duration, and all subscales of the Quality of Life in Epilepsy Inventory (QOLIE-31). There was also a statistically significant association between anxiety and educational level, employment status, history of psychiatric disorders, epilepsy duration, and all subscales of QOLIE-31. The mean overall QOLIE-31 score was 60.21 ± 20.19, with educational level and employment status, among other factors, being significantly associated with QOLIE-31. **Conclusions:** Depression and anxiety are prevalent among epileptic patients, requiring routine screening. Supporting education and employment among epileptic patients also appears to be crucial. Strategies to improve QoL among this population should be developed.

## 1. Introduction

Epilepsy is a chronic neurological disorder characterized by recurrent seizures that affect various aspects of an individual’s life [1,2]. Such seizures manifest as involuntary movements involving parts or the entirety of the body and could lead to transient neurological symptoms [1,2]. Symptoms vary from motor disturbance, loss of awareness, emotional disturbance, to sensory changes [3]. The World Health Organization (WHO) estimates that five million new cases are diagnosed each year, and 50 million individuals worldwide suffer from epilepsy [4]. The prevalence of epilepsy in Saudi Arabia is 3.96 per 1000 [5].

Depression is one of the common psychiatric disorders [6]. According to the Diagnostic and Statistical Manual of Mental Disorders 5th edition (DSM-5), a major depressive episode is defined as having five or more symptoms, out of nine, for two weeks or more, that represent a change from the previous level of functioning [7]. According to the WHO, about 280 million people worldwide are affected by depression, and 3.8% of people are affected during their lifetime [8]. Furthermore, depression is one of the most common causes of suicide [8]. In Saudi Arabia, the prevalence of major depressive disorder (MDD) is 12.7% [9]. Studies indicate that treating depression can directly improve epilepsy management outcomes [10,11].

Anxiety is also one of the prevalent psychiatric disorders [12]. According to DSM-5, generalized anxiety disorder (GAD) is defined as excessive anxiety and worry occurring more days than not for at least 6 months [7]. The WHO estimates that 301 million people experienced anxiety in 2019 [13]. In Saudi Arabia, the prevalence of GAD is estimated to be around 12.4% [9].

There is a strong association between epilepsy and depression. Research shows that compared to the general population, those with epilepsy have a higher prevalence of depression [14,15]. A study on depression in patients with newly diagnosed epilepsy conducted in Strasbourg, France, revealed that 11% of the participants reported depression [16]. Another study conducted in Barcelona, Spain, found that almost half of epileptic patients had depression [17]. In Saudi Arabia, one study demonstrated that half of epileptic patients have depression [18]. Another Saudi study in Taif indicated that almost three-quarters of epileptic patients have depression [19]. Depression affects many facets of life for those who have epilepsy, presenting major challenges to seizure control and general quality of life. For instance, studies indicate that depression is associated with more frequent and severe seizures [10,20]. Despite this concerning correlation, about one-fifth of people with depression remain untreated, and about one-third of epilepsy cases are undetected [11]. Moreover, depression was consistently strongly correlated with low self-efficacy in preventing seizures, negative attitudes regarding epilepsy, and low self-esteem [21].

Anxiety also has a strong association with epilepsy. Anxiety was shown to be a major problem for epileptic patients in a study conducted in Barcelona, Spain, which found that about half of epileptic patients had GAD [17]. In Saudi Arabia, 15% to 25% of the participants in one study were found to have social anxiety [22]. Anxiety disorders need to be treated, as they raise the risk of a lower quality of life and can have serious consequences [23].

Additionally, the quality of life of individuals with epilepsy is greatly affected by a number of factors, the most notable of which are seizure frequency and severity, anxiety, depression, and adverse effects from antiepileptic medications [19,22,24,25,26,27,28]. Numerous studies have shown that in people with epilepsy, psychiatric illnesses and quality of life are negatively correlated [10,11,20,22,24,25,27,28].

This study aims to assess the prevalence of depression and anxiety symptoms in epileptic patients at King Khalid University Hospital (KKUH) and to assess their quality of life.

## 2. Materials and Methods

### 2.1. Study Design, Setting, and Participants

This is a cross-sectional study that was conducted among patients attending the Neurology Clinics and the Neuropsychiatry Clinic at KKUH in Riyadh, Saudi Arabia.

Concerning the targeted population, the study included all patients diagnosed with epilepsy in the aforementioned setting. More specifically, the inclusion criteria entailed adult patients, males and females, who are 18 years or older with a diagnosis of epilepsy at KKUH. The exclusion criteria entailed patients who are younger than 18 years old and those with communication barriers.

The number of epileptic patients was extrapolated by the information technology (IT) department from eSiHi (the electronic medical record system used in KKUH) using the keyword “epilepsy”, resulting in 1845 patients. We calculated the sample size using the Raosoft website, with a confidence level of 95% and a margin of error of 5%, resulting in an estimated sample size of 319 patients. In the study, a convenience sampling technique was used to recruit participants.

In the study, the authors collected the data remotely (virtually) from the participants, using the digital form of the study tool (which was created using SurveyMonkey.com). The virtual approach was achieved by obtaining the list of the targeted population’s file numbers (and hence their contact information) from the IT department after receiving the Institutional Review Board approval for the study. Thus, using the list from the IT department, we contacted the participants virtually to invite them to participate in the research and to collect their data. Data collection took place between September and December 2024.

### 2.2. Study Instruments

The study instrument consisted of a questionnaire developed by the research team, the Arabic version of the Hospital Anxiety and Depression Scale (HADS), and the Arabic version of the Quality of Life in Epilepsy Inventory (QoLIE-31).

The questionnaire was divided into two parts. The first part aimed to assess sociodemographic factors including age, gender, nationality, employment, education level, marital status, and history of mental illness. The second part concerned the clinical features of epilepsy, including duration, whether there were any seizures in the last 6 months, family history of epilepsy, the number of medications the participants are currently on, and the medication type.

The HADS consists of 14 items: seven items assessing anxiety symptoms and seven assessing depression symptoms [29]. The items are rated on a 4-item Likert-type scale from 0 to 3 with a maximum score of 21 for each subscale [29]. We used the Arabic validated version of the HADS, which has a Cronbach’s alpha of 0.78 and 0.88 for the anxiety and depression subscales, respectively [30,31]. Permission was granted to use the Arabic version of the scale in our study [32].

The QoLIE-31 scale addresses key areas of concern for individuals with epilepsy, providing a means to evaluate both epilepsy-specific issues and broader aspects of quality of life [33]. QoLIE-31 contains 31 items divided into seven subscales, assessing different domains [33]. The subscales are seizure worry, overall quality of life, emotional well-being, cognitive functioning, antiepileptic medication effects, energy/fatigue, and social functioning [33]. Answers for each item are converted to a scale of 0–100 points, with higher values indicating better quality of life. The points are then summed for each subscale, and a final score from 0 to 100 is derived from the total sums. Higher values mean better quality of life [33]. We used the Arabic validated version of the QoLIE-31 scale, which has a Cronbach’s alpha of 0.993 [34]. The QoLIE-31 scale is owned by the RAND Corporation, and according to their policy, all tools that they own are in the public domain and available to be used [35]. Permission was also granted from the authors of the validation study of the Arabic version.

### 2.3. Descriptive Statistics

Statistical analyses were performed using R software version 4.4.0 (R Foundation for Statistical Computing, Vienna, Austria). The internal consistency of the questionnaire scales was measured to test the reliability using Cronbach’s alpha coefficient, and an alpha value equal to or greater than 0.7 was considered satisfactory. Continuous variables were tested for normality with the Shapiro test and are presented as mean ± standard deviation (SD) and range. Categorical variables were presented as frequency (percentage). We investigated the correlation between the scores of the HADS scales with different quality-of-life subscales through the Spearman correlation coefficient test. Univariate logistic regression was carried out to evaluate potential predictors of depression and anxiety. Univariate and multivariate linear regression analysis was conducted to assess potential predictors of quality of life (dependent variable: QOLIE-31 overall score). A two-tailed *p*-value of less than 0.05 was statistically significant.

### 2.4. Ethical Consideration

In the study’s instrument tool, we indicated the title and the purpose of the study, emphasizing that participating in the study was voluntary, provided the name and contact information of the primary investigator, and gave assurances regarding the confidentiality of the information obtained. Participants were also informed and assured that all data collected would be anonymous and confidential and would be used for research-related purposes only. The study was reviewed and approved by the Institutional Review Board at the College of Medicine at King Saud University (Research Project No. E-24-8954).

## 3. Results

Four-hundred adult patients participated in the study. Of those, nearly one-third (31.25%) were older than 45, 27.25% were aged between 25 and 35 years, and 53% (*n* = 212) were female. Most of the participants (98.00%) were Saudi, and nearly half (46.75%) were married. Approximately half of the respondents had a bachelor’s degree (43.25%, *n* = 173), and 50.0% (*n* = 200) were unemployed. Sixty-five participants (16.25%) had been diagnosed with psychiatric disorders, and 62 participants (15.5%) had a family history of psychiatric disorders. More than a third (40.50%) had a duration of seizures of more than 15 years, and 213 participants (53.25%) were receiving monotherapy. More than half (54.75%) of the participants were taking levetiracetam, followed by 107 (26.75%) who were taking carbamazepine. Seizures were found in 154 participants (38.5%) within the last 6 months, and a family history of seizure was found in 86 participants (21.5%). An overview of the relevant clinical and sociodemographic characteristics of the participants is provided in Table 1.

### 3.1. The HADS Scales

The internal consistency of the HADS as measured by Cronbach’s alpha coefficient was 0.78 for the depression subscale and 0.84 for the anxiety subscale, indicating satisfactory reliability. The mean scores were 7.18 ± 4.61 for the HADS depression subscale and 6.60 ± 4.64 for the HADS anxiety subscale. According to the HADS scores (8 or above), 193 (48.25%) patients had symptoms of depression, and 159 (39.75%) had symptoms of anxiety. The prevalence of depression in epileptic patients seems to be higher than that of anxiety. Statistics of the HADS scale are presented in Table 2.

### 3.2. QOLIE-31

The quality of life of patients with epilepsy was assessed using QOLIE-31, where higher scores reflect better quality of life. The internal consistency of QOLIE-31, as measured by Cronbach’s alpha coefficient, was 0.94. Table 3 shows the mean and SD scores of the QOLIE-31 subscales. The mean of the QOLIE-31 overall score was 60.21 ± 20.19. The highest mean score was for the overall quality of life subscale, 70.29 ± 22.61, and the lowest was for the medication effects subscale, 47.85 ± 34.22. The mean of the subjective overall health subscale was 70.86 ± 24.56.

### 3.3. Correlations Between Quality of Life and Anxiety and Depression 

The HADS depression scores are inversely correlated with all subscales of QOLIE-31, with coefficients ranging between *r* = −0.29 (*p* < 0.001) for medication effects and *r* = −0.71 (*p* < 0.001) for emotional well-being. Moreover, the HADS anxiety scores were inversely correlated with all subscales of QOLIE-31, with coefficients ranging between *r* = −0.39 (*p* < 0.001) for medication effects and *r* = −0.79 (*p* < 0.001) for emotional well-being. Correlations between anxiety and depression, and quality of life, are presented in Table 4.

### 3.4. Factors Associated with the HADS Scales

The univariable logistic regression revealed that educational level, employment status, history of psychiatric disorders, epilepsy duration, and all subscales of QOLIE-31 were significantly associated with depression. Educated individuals were less likely to have depression than the uneducated (OR = 0.20; *p* = 0.001) for those with high school/lesser, (OR = 0.10; *p* < 0.001) for bachelor’s degree, and (OR = 0.18; *p* = 0.003) for postgraduate. Similarly, unemployed patients were more likely to have depression than employed patients (OR = 0.41; *p* < 0.001), and patients who had a history of psychiatric disorders were 4.48 times (OR = 4.48; *p* < 0.001) more likely to have depression. Patients who had epilepsy for 5–10 years were less likely to have depression than patients with a duration of less than 5 years (OR = 0.55; *p* = 0.049). Patients were less likely to have depression with high seizure worry (OR = 0.98; *p* < 0.001), high overall quality of life (OR = 0.94; *p* < 0.001), high emotional well-being (OR = 0.92; *p* < 0.001), high energy/fatigue (OR = 0.93; *p* < 0.001), high cognitive functioning (OR = 0.95; *p* < 0.001), high medication effects (OR = 0.98; *p* < 0.001), and high social functioning (OR = 0.95; *p* < 0.001) (Table 5).

The univariable logistic regression revealed that educational level, employment status, history of psychiatric disorders, epilepsy duration, and all subscales of QOLIE-31 were significantly associated with anxiety. Patients with a bachelor’s degree were less likely to have anxiety than uneducated patients (OR = 0.34; *p* = 0.002). Employed patients were less likely to have anxiety than unemployed patients (OR = 0.55; *p* = 0.006), and patients who had a history of psychiatric disorders were 3.69 times (OR = 3.69; *p* < 0.001) more likely to have anxiety. Similarly, patients who had epilepsy for less than 5 years were more likely to have anxiety than those who had epilepsy for 5–10 years (OR = 0.44; *p* = 0.008), 10–15 years (OR = 0.49; *p* = 0.038), or more than 15 years (OR = 0.56; *p* = 0.023). Patients who had seizures in the last 6 months were 2.09 times (OR = 2.09; *p* < 0.001) more likely to have anxiety. Patients were less likely to have anxiety with high seizure worry (OR = 0.97; *p* < 0.001), high overall quality of life (OR = 0.94; *p* < 0.001), high emotional well-being (OR = 0.90; *p* < 0.001), high energy/fatigue (OR = 0.94; *p* < 0.001), high cognitive functioning (OR = 0.96; *p* < 0.001), high medication effects (OR = 0.98; *p* < 0.001), and high social functioning (OR = 0.95; *p* < 0.001) (Table 5).

### 3.5. Factors Associated with Quality of Life (QOLIE-31)

The mean overall QOLIE-31 score was 60.21 ± 20.19 (range: 8.95–100). The univariate analysis indicated that a high QOLIE-31 score was more associated with patients who had bachelor’s degrees than uneducated patients (β = 11.65; *p* = 0.001) and more associated with employed patients than unemployed patients (β = 6.54; *p* = 0.002). Additionally, the univariate analysis indicated that a low QOLIE-31 score was associated with patients who had a history of psychiatric disorders (β = −15.42; *p* < 0.001), a medication number of 2 compared to 1 (β = −5.95; *p* < 0.008), a medication number of ≥3 compared to 1 (β = −10.78; *p* < 0.001), seizures in the last 6 months (β = −10.00; *p* < 0.001), and family history of seizure (β = −5.71; *p* < 0.020). Similarly, patients who had depression (β = −3.16; *p* < 0.001) and those who had anxiety (β = −3.23; *p* < 0.001) according to HADS scale scores were significantly associated with a low QOLIE-31 score (Table 6).

The multivariate linear regression model indicated that the factors significantly associated with a low QOLIE-31 score were medication number, seizures in the last 6 months, family history of seizures, HADS depression, and anxiety. Patients who had ≥3 medications compared to those who had 1 medication (β = −4.14; *p* = 0.028) were associated with a low QOLIE-31 score. Patients with seizures in the last 6 months (β = −4.47; *p* = 0.001) and a family history of seizure (β = −3.08; *p* = 0.041) were associated with a low QOLIE-31 score. Similarly, patients who had depression (β = −2.02; *p* < 0.001) and those who had anxiety (β = −1.78; *p* < 0.001) according to HADS scales scores were significantly associated with a low QOLIE-31 score (Table 6).

## 4. Discussion

This study aims to estimate the prevalence of depression and anxiety among epileptic patients, assess their quality of life, and explore possible risk factors that may be associated with epilepsy and the overall quality of life in these patients. The study’s findings can contribute to a deeper understanding of depression and anxiety in individuals with epilepsy in the Saudi Arabian context. Furthermore, its results could provide valuable insights for clinicians to develop more targeted and effective interventions, ultimately improving the care and quality of life for epileptic patients.

Almost half of the participants demonstrated some degree of depression. This finding is in line with a study conducted in Barcelona, Spain, indicating that almost half of patients with epilepsy (PWE) were found to have depression, as measured using the HADS-D scale [17]. However, a study conducted in Thailand found that one-fifth of PWE had depression [36]. The lower prevalence in that study could be attributed to its inclusion criteria, which focused only on individuals aged 15–50 years, as well as the smaller sample compared to our study. In our results, half of the participants showed varying degrees of depression. This included a moderate level of depression in approximately one-fifth of participants and a severe level in about 6%, according to HADS-A. In Saudi Arabia, a study was conducted in Taif city using the Patient Health Questionnaire-9 (PHQ-9) score [19]. In that study, about three-quarters of participants exhibited depression, with varying degrees of severity. Among them, around one-tenth were identified as having severe depression, while approximately 13% were classified as moderately severe [19]. The lower percentage of depression observed in our study compared to the Taif study might be at least partially due to the difference in the screening tool employed to evaluate depressive symptoms. Nevertheless, the findings of our study highlight the significant prevalence of depressive symptoms among PWE, underscoring the need for routine depression screening in this particular population. In terms of anxiety, among our study’s participants, about 40% of PWE appeared to have anxiety, which aligns with the results of a study conducted in Brazil [37]. Such a finding indicates a high percentage of anxiety among PWE, emphasizing the need to conduct anxiety screening in this particular population.

In our study, several factors were associated with depression and anxiety in PWE. Our findings indicated that educated patients were less likely to have depression and anxiety than uneducated patients. With regard to depression, our findings are consistent with a study conducted in Northwest Ethiopia, which found a significant association between educational status and depression [38]. Furthermore, our results suggested that educated patients had better QOLIE-31 scores. This finding corresponds to a study conducted in the Qassim Region of Saudi Arabia [39]. The authors of that Saudi study attributed this to educated patients’ improved understanding of disease, awareness of triggers, and seizure management. Such a finding indicates the importance of taking patients’ education level into account when planning and approaching their treatment.

Moreover, in our study, employment was associated with better quality of life and seemed to be protective against depression and anxiety, which aligns with other studies’ findings [40,41]. Such a finding could be explained by the economic pressure caused by unemployment [42]. This finding highlights the value of employment in epileptic patients, as it enhances chances for social support and personal satisfaction.

Participants with a personal history of psychiatric disorders in our study were found to have greater odds of developing depression and anxiety compared to those without a personal history of psychiatric disorders. This finding is consistent with a study conducted in Strasbourg, France, which found that individuals with a psychiatric history are more likely to present with anxiety and depressive symptoms at the time of an epilepsy diagnosis [16]. This indicates that psychiatric comorbidities, including depression and anxiety, should be routinely assessed in epileptic patients, as they may exacerbate the condition.

Participants with a shorter duration of epilepsy in our study were more likely to experience depression and anxiety; in contrast, those with a longer duration reported lower levels of such symptoms. This finding aligns with a study conducted in Australia, which showed that significant symptoms of depression and anxiety frequently occur during the initial seizure or shortly after an epilepsy diagnosis [43]. Another study also showed that mood disorders are prevalent among patients with both newly diagnosed and chronic epilepsy [44]. Based on these findings, early detection of depression and anxiety in PWE is crucial.

In our study, participants who experienced a seizure in the last 6 months were likely to suffer from anxiety. Interestingly, our data showed that individuals who expressed high levels of concern or worries about their seizures had lower rates of depression and anxiety. We hypothesize that this might be because their heightened concern about their health led to greater adherence to medications, as well as more consistent follow-ups and referrals. In contrast, a study conducted in Wuhan, China, concluded that worrying about seizures is a significant risk factor for elevated scores on depression and anxiety [45]. The difference between that study’s findings and ours might be attributable to cultural variations and differences in methodology, as the Wuhan study used the PHQ-9 score.

Our study’s findings indicate that there is no significant association between depression or anxiety and variables such as age, gender, marital status, family history of psychiatric disorders, and family history of seizures. This finding is consistent with a study conducted in Australia, which demonstrated a weak relationship between sociodemographic factors and the prevalence of depression and anxiety in PWE [43].

Our study found low QOLIE-31 scores in patients receiving multiple antiepileptic medications (i.e., polytherapy). This finding aligns with studies conducted in the Qassim Region of Saudi Arabia and another conducted in Nepal [39,41]. This finding indicates that polytherapy should be avoided as much as possible, as it causes more side effects, which can worsen patients’ quality of life. Moreover, our data showed a low QOLIE-31 score in participants with a positive family history of epilepsy, which aligns with the study conducted in Nepal [41]. This finding is clinically important, as these patients may need personalized care strategies, such as a comprehensive family history analysis, which includes identifying effective medications for family members, as well as recognizing exacerbating and supportive factors.

Our study found that patients with anxiety have a more negative correlation in the “seizure worry” and “medication effects” subscales of QOLIE-31 compared to depressed participants. Our hypothesis for these findings is that anxiety can amplify the emotional and psychological distress associated with seizures, leading to more profound negative correlations in the “seizure worry” subscale. In the “medication effects” subscale, our explanation for this finding is that anxious patients may be more sensitive to side effects and the perceived ineffectiveness of their medications, resulting in a stronger negative correlation. The perceived anxiety, depression, and reduced quality of life are well described in the literature among PWE [17].

Our study has strengths and limitations. Regarding its strengths, we used validated scales, which add credibility and reliability to our findings. Another strength is the number of participants who were enrolled in the study. Furthermore, examining quality of life, depression, and anxiety concurrently in the same study provides a more comprehensive picture of patients’ experiences.

Our study also has certain limitations. Our study was limited to one hospital in Riyadh, which might affect the generalizability of the results. Moreover, recall bias might have influenced the findings due to the use of some retrospective items in our study instrument. Another specific limitation of the study tool was that the research team asked the participants whether or not they have mood disorders in general rather than asking specifically about depression. Future research could address this limitation by asking specifically about depression and, hence, reflect better comparisons between subjective and objective findings. Also, in this study, the research team did not collect information about the use of antidepressants among the participants. Collecting such information in future research could help examine the association between antidepressants and epilepsy’s outcome and the subsequent risk of depression and anxiety among PWE, enriching the understanding of associated factors. Last, future research should adopt a longitudinal approach, incorporating formal psychiatric assessment and neuroimaging.

## 5. Conclusions

This cross-sectional study aimed to assess the prevalence of depressive and anxiety symptoms among epileptic patients. The results showed that depression and anxiety are prevalent among PWE. Tactics to mitigate the negative consequences of this finding in the Saudi context should be developed. One way to achieve such an outcome could be to emphasize and support integrated care, which entails collaborative efforts between mental health and neurology professionals, resulting in more comprehensive and favorable epilepsy outcomes. Another meaningful way is to develop and support screening programs for anxiety and depression among PWE. Our study also found that being educated and employed can help reduce the risk of depression and anxiety among PWE. As such, supporting education and employment among PWE is needed. Although this study examined the epilepsy diagnosis, it is worth mentioning that exploring depression and anxiety among other neurological conditions, such as stroke, is crucial [46]. Furthermore, examining the effects of various antiseizure medications on behavior, including neuropsychiatric manifestations, is essential [47]. Additionally, examining more in-depth common comorbidities of depression and anxiety, such as attention deficit hyperactivity disorder, in relation to these illnesses and to epilepsy would enrich our understanding of the topic.

## Figures and Tables

**Table 1:** Clinical and sociodemographic characteristics of the participants.

	*N* = 400
Age	
18–25	72 (18.00%)
25–35	109 (27.25%)
35–45	94 (23.50%)
>45	125 (31.25%)
Gender	
Male	188 (47.00%)
Female	212 (53.00%)
Nationality	
Saudi	392 (98.00%)
Non-Saudi	8 (2.00%)
Marital status	
Single	173 (43.25%)
Married	187 (46.75%)
Divorced	24 (6.00%)
Widower	16 (4.00%)
Educational level	
Uneducated	40 (10.00%)
High school/lesser	159 (39.75%)
Bachelor	173 (43.25%)
Postgraduate	28 (7.00%)
Employment status	
Student	37 (9.25%)
Unemployed	200 (50.00%)
Employed	163 (40.75%)
Psychiatric disorders	65 (16.25%)
Psychiatric disorders	
Mood disorders	31 (7.75%)
Anxiety disorders	30 (7.50%)
Psychotic disorders	6 (1.50%)
Others	13 (3.25%)
Family history of psychiatric disorders	62 (15.50%)
Duration of epilepsy	
<5 years	102 (25.50%)
5–10 years	81 (20.25%)
10–15 years	55 (13.75%)
>15 years	162 (40.50%)
Number of anti-seizure medications	
1	213 (53.25%)
2	128 (32.00%)
≥3	59 (14.75%)
Anti-seizure medications	
Lamotrigine	67 (16.75%)
Levetiracetam	219 (54.75%)
Carbamazepine	107 (26.75%)
Topiramate	38 (9.50%)
Phenytoin	11 (2.75%)
Valproic acid	82 (20.50%)
Others	107 (26.75%)
Seizure in the last 6 months	154 (38.50%)
Family history of seizure	86 (21.50%)

**Table 2:** Statistics of HADS scales.

	Cronbach’s Alpha	Mean ± SD	Min–Max	*N* (%)	
HADS depression	0.78	7.18 ± 4.61	0–21	Normal	207 (51.75%)
				Mild	95 (23.75%)
				Moderate	74 (18.50%)
				Severe	24 (6.00%)
HADS anxiety	0.84	6.60 ± 4.64	0–19	Normal	241 (60.25%)
				Mild	73 (18.25%)
				Moderate	60 (15.00%)
				Severe	26 (6.50%)

**Table 3:** Statistics of quality-of-life subscales in QOLIE-31.

	Number of Items	Cronbach’s Alpha	Mean ± SD	Min–Max
Seizure worry	5	0.89	49.91 ± 32.18	0–100
Overall quality of life	2	0.73	70.29 ± 22.61	0–100
Emotional well-being	5	0.84	61.28 ± 21.85	0–100
Energy/Fatigue	4	0.81	52.69 ± 22.37	0–100
Cognitive functioning	6	0.87	56.99 ± 25.74	0–100
Medication effects	3	0.84	47.85 ± 34.22	0–100
Social functioning	5	0.80	66.88 ± 26.36	0–100
Subjective overall health	1		70.86 ± 24.56	0–100
QOLIE-31 overall score	30	0.94	60.21 ± 20.19	8.95–100

**Table 4:** Correlation of HADS scales with quality-of-life subscales in QOLIE-31.

	HADS Depression	HADS Anxiety
Seizure worry	−0.31 *	−0.44 *
Overall quality of life	−0.62 *	−0.61 *
Emotional well-being	−0.71 *	−0.79 *
Energy/Fatigue	−0.67 *	−0.61 *
Cognitive functioning	−0.58 *	−0.56 *
Medication effects	−0.29 *	−0.39 *
Social functioning	−0.60 *	−0.61 *
QOLIE-31 overall score	−0.72 *	−0.74 *

* *p* < 0.05. QOLIE-31, Quality of Life in Epilepsy-31; HADS, Hospital Anxiety Depression Scale.

**Table 5:** Univariate logistic regression analysis of variables associated with depression and anxiety among epileptic patients.

Variable	Depression		Anxiety	
	OR (95% CI)	*p*-Value	OR (95% CI)	*p*-Value
Age (vs. >45)				
18–25	0.63 (0.34–1.12)	0.120	1.40 (0.78–2.53)	0.258
25–35	0.90 (0.54–1.50)	0.681	0.89 (0.52–1.52)	0.664
35–45	1.12 (0.65–1.91)	0.682	1.28 (0.74–2.22)	0.369
Gender (vs. female)				
Male	1.05 (0.71–1.56)	0.796	0.72 (0.48–1.08)	0.114
Nationality (vs. non-Saudi)				
Saudi	0.55 (0.11–2.28)	0.422	1.10 (0.27–5.43)	0.896
Marital status (vs. single)				
Married	0.92 (0.61–1.39)	0.700	0.70 (0.45–1.07)	0.097
Divorced	1.48 (0.63–3.61)	0.371	1.92 (0.81–4.68)	0.140
Widower	1.06 (0.37–3.00)	0.912	1.76 (0.63–5.14)	0.283
Educational level (vs. uneducated)				
High school/lesser	0.20 (0.07–0.47)	0.001 *	0.57 (0.28–1.14)	0.112
Bachelor	0.10 (0.03–0.23)	<0.001 *	0.34 (0.16–0.67)	0.002 *
Postgraduate	0.18 (0.05–0.53)	0.003 *	0.64 (0.24–1.69)	0.369
Employment status (vs. unemployed)				
Employed	0.41 (0.27–0.63)	<0.001 *	0.55 (0.35–0.84)	0.006 *
Student	0.54 (0.26–1.09)	0.089	1.02 (0.50–2.06)	0.960
History of psychiatric disorders	4.48 (2.47–8.55)	<0.001 *	3.69 (2.13–6.56)	<0.001 *
Family history of psychiatric disorders	1.73 (1.00–3.02)	0.052	1.41 (0.81–2.42)	0.220
Duration of epilepsy (vs. <5 years)				
5–10 years	0.55 (0.30–0.99)	0.049 *	0.44 (0.24–0.80)	0.008 *
10–15 years	0.99 (0.51–1.92)	0.980	0.49 (0.24–0.95)	0.038 *
>15 years	0.85 (0.51–1.39)	0.509	0.56 (0.34–0.92)	0.023 *
Number of anti-seizure medications (vs. one therapy)				
2	1.07 (0.69–1.66)	0.768	1.25 (0.80–1.96)	0.334
≥3	1.60 (0.89–2.88)	0.116	1.71 (0.95–3.06)	0.072
Seizure in the last 6 months	1.22 (0.82–1.83)	0.335	2.09 (1.39–3.17)	<0.001 *
Family history of seizure	0.81 (0.50–1.31)	0.395	1.26 (0.78–2.04)	0.343
Seizure worry	0.98 (0.97–0.99)	<0.001 *	0.97 (0.97–0.98)	<0.001 *
Overall quality of life	0.94 (0.92–0.95)	<0.001 *	0.94 (0.93–0.95)	<0.001 *
Emotional well-being	0.92 (0.90–0.93)	<0.001 *	0.90 (0.88–0.92)	<0.001 *
Energy/Fatigue	0.93 (0.91–0.94)	<0.001 *	0.94 (0.92–0.95)	<0.001 *
Cognitive functioning	0.95 (0.94–0.96)	<0.001 *	0.96 (0.95–0.97)	<0.001 *
Medication effects	0.98 (0.98–0.99)	<0.001 *	0.98 (0.97–0.98)	<0.001 *
Social functioning	0.95 (0.94–0.96)	<0.001 *	0.95 (0.94–0.96)	<0.001 *

* *p* < 0.05. CI, confidence interval.

**Table 6:** Univariate and multivariate linear regression analysis of variables associated with QOLIE-31 overall score among epileptic patients.

Variable	Univariate		Multivariate	
	Coefficient β (95% CI)	*p*-Value	Coefficient β (95% CI)	*p*-Value
Age (vs. >45)				
18–25	−0.14 (−6.02 to 5.74)	0.963	−1.83 (−7.07 to 3.40)	0.491
25–35	−1.43 (−6.64 to 3.78)	0.590	−1.73 (−5.42 to 1.97)	0.360
35–45	−3.23 (−8.66 to 2.20)	0.243	−0.54 (−3.96 to 2.87)	0.755
Gender (vs. female)				
Male	1.45 (−2.52 to 5.43)	0.473	0.49 (−2.16 to 3.14)	0.717
Nationality (vs. non-Saudi)				
Saudi	1.93 (−12.26 to 16.13)	0.789	−0.16 (−8.59 to 8.27)	0.971
Marital status (vs. single)				
Married	2.85 (−1.33 to 7.02)	0.181	0.75 (−2.63 to 4.12)	0.664
Divorced	−5.37 (−13.99 to 3.26)	0.222	−2.54 (−8.06 to 2.98)	0.366
Widower	−2.59 (−12.94 to 7.76)	0.623	0.56 (−6.60 to 7.72)	0.878
Educational level (vs. uneducated)				
High school/lesser	5.70 (−1.22 to 12.62)	0.106	−3.69 (−8.40 to 1.03)	0.125
Bachelor	11.65 (4.78 to 18.51)	0.001 *	−4.75 (−9.79 to 0.29)	0.065
Postgraduate	7.38 (−2.26 to 17.03)	0.133	−5.98 (−12.53 to 0.58)	0.074
Employment status (vs. unemployed)				
Employed	6.54 (2.40 to 10.69)	0.002 *	−1.31 (−4.41 to 1.78)	0.404
Student	6.64 (−0.38 to 13.67)	0.064	3.54 (−1.62 to 8.70)	0.178
History of psychiatric disorders	−15.42 (−20.59 to −10.26)	<0.001 *	−1.78 (−5.20 to 1.64)	0.306
Family history of psychiatric disorders	−3.32 (−8.80 to 2.16)	0.234	−0.71 (−4.01 to 2.59)	0.672
Duration of epilepsy (vs. <5 years)				
5–10 years	5.51 (−0.38 to 11.40)	0.067	0.96 (−2.60 to 4.52)	0.596
10–15 years	6.48 (−0.14 to 13.10)	0.055	3.80 (−0.22 to 7.82)	0.064
>15 years	3.21 (−1.79 to 8.22)	0.208	2.43 (−0.80 to 5.65)	0.139
Number of anti-seizure medications (vs. one therapy)				
2	−5.95 (−10.31 to −1.59)	0.008 *	−2.04 (−4.91 to 0.83)	0.163
≥3	−10.78 (−16.52 to −5.05)	<0.001 *	−4.14 (−7.82 to −0.46)	0.028 *
Seizure in the last 6 months	−10.00 (−13.97 to −6.04)	<0.001 *	−4.47 (−7.13 to −1.81)	0.001 *
Family history of seizure	−5.71 (−10.51 to −0.91)	0.020 *	−3.08 (−6.03 to −0.13)	0.041 *
HADS depression	−3.16 (−3.46 to −2.86)	<0.001 *	−2.02 (−2.38 to −1.66)	<0.001 *
HADS anxiety	−3.23 (−3.52 to −2.94)	<0.001 *	−1.78 (−2.13 to −1.44)	<0.001 *

* *p* < 0.05. CI, confidence interval. HADS, Hospital Anxiety Depression Scale.

## Data Availability

The raw data supporting the conclusions of this article will be made available by the authors upon request.

## References

[1] Fisher R.S., Acevedo C., Arzimanoglou A., Bogacz A., Cross J.H., Elger C.E., Engel J., Forsgren L., French J.A., Glynn M. (2014). ILAE official report: A practical clinical definition of epilepsy. Epilepsia.

[2] Fisher R.S., van Emde Boas W., Blume W., Elger C., Genton P., Lee P., Engel J. (2005). Epileptic seizures and epilepsy: Definitions proposed by the International League Against Epilepsy (ILAE) and the International Bureau for Epilepsy (IBE). Epilepsia.

[3] Blume W.T., Lüders H.O., Mizrahi E., Tassinari C., van Emde Boas W., Engel J. (2001). Glossary of descriptive terminology for ictal semiology: Report of the ILAE task force on classification and terminology. Epilepsia.

[4] World Health Organization *Epilepsy* 2023. https://www.who.int/news-room/fact-sheets/detail/epilepsy#:~:text=Globally%2C%20an%20estimated%205%20million,diagnosed%20with%20epilepsy%20each%20year.

[5] Idris A., Alabdaljabar M.S., Almiro A., Alsuraimi A., Dawalibi A., Abduljawad S., AlKhateeb M. (2021). Prevalence, incidence, and risk factors of epilepsy in arab countries: A systematic review. Seizure.

[6] Chand S.P., Arif H. (2025). Depression. [Updated 2023 Jul 17]. StatPearls.

[7] American Psychiatric Association (2013). Diagnostic and Statistical Manual of Mental Disorders.

[8] World Health Organization (2023). Depressive disorder (Depression). https://www.who.int/news-room/fact-sheets/detail/depression#:~:text=Women%20are%20more%20likely%20to,world%20have%20depression%20(1).

[9] Alhabeeb A.A., Al-Duraihem R.A., Alasmary S., Alkhamaali Z., Althumiri N.A., BinDhim N.F. (2023). National screening for anxiety and depression in Saudi Arabia 2022. Front. Public Health.

[10] Johnson E.K., Jones J.E., Seidenberg M., Hermann B.P. (2004). The relative impact of anxiety, depression, and clinical seizure features on health-related quality of life in epilepsy. Epilepsia.

[11] Boylan L.S., Flint L.A., Labovitz D.L., Jackson S.C., Starner K., Devinsky O. (2004). Depression but not seizure frequency predicts quality of life in treatment-resistant epilepsy. Neurology.

[12] Kessler R.C., Berglund P., Demler O., Jin R., Merikangas K.R., Walters E.E. (2005). Lifetime prevalence and age-of-onset distributions of DSM-IV disorders in the National Comorbidity Survey Replication. Arch. Gen. Psychiatry.

[13] World Health Organization (2023). Any. Anxiety Disord.

[14] Engidaw N.A., Bacha L., Kenea A. (2020). Prevalence of depression and associated factors among epileptic patients at Ilu Ababore zone hospitals, South West Ethiopia, 2017: A cross-sectional study. Ann. Gen. Psychiatry.

[15] Edeh J., Toone B.K. (1985). Antiepileptic therapy, folate deficiency, and psychiatric morbidity: A general practice survey. Epilepsia.

[16] Forthoffer N., Tarrada A., Brissart H., Maillard L., Hingray C. (2021). Anxiety and Depression in Newly Diagnosed Epilepsy: A Matter of Psychological History?. Front. Neurol..

[17] Rocamora R., Chavarría B., Pérez E., Pérez-Enríquez C., Barguilla A., Panadés-de Oliveira L., Principe A., Zucca R. (2021). Mood Disturbances, Anxiety, and Impact on Quality of Life in Patients Admitted to Epilepsy Monitoring Units. Front. Neurol..

[18] Albalawi R.S.A., Alanzi S.M.O., Alharthe A.F.H., Atawi S.H.S., Albalawi R.M.D., Alanazi H.A.S., Alsayed M.S.A., Zubair M. (2023). Quantitative Cross-Sectional Study About the Prevalence of Depression Among Epileptic Patients in Saudi Arabia. Cureus.

[19] Mubaraki A.A., Sibyani A.K., Alkhawtani R.A., Alqahtani B.G., Abu Alaynayn F.K. (2021). Prevalence of depression among epileptic patients in Taif City, Saudi Arabia. Neurosciences (Riyadh).

[20] Agrawal N., Bird J.S., von Oertzen T.J., Cock H., Mitchell A.J., Mula M. (2016). Depression correlates with quality of life in people with epilepsy independent of the measures used. Epilepsy Behav..

[21] Temple J., Fisher P., Davies C., Millar C., Gemma Cherry M. (2023). Psychosocial factors associated with anxiety and depression in adolescents with epilepsy: A systematic review. Epilepsy Behav..

[22] Alkoblan F.I., Alsoadan M.M., Alhajri A.A., Almousa M.M., Alsalamah F.S., Kazi A., Bashiri F.A., Aljafen B. (2023). Social Anxiety, Social Support, and Quality of Life in Patients With Epilepsy at a Tertiary Care Hospital in Saudi Arabia. Cureus.

[23] Vazquez B., Devinsky O. (2003). Epilepsy and anxiety. Epilepsy Behav..

[24] Nigussie K., Lemma A., Sertsu A., Asfaw H., Kerebih H., Abdeta T. (2021). Depression, anxiety and associated factors among people with epilepsy and attending outpatient treatment at primary public hospitals in northwest Ethiopia: A multicenter cross-sectional study. PLoS ONE.

[25] Tsamakis K., Teagarden D.L., Villarreal H.K., Morton M.L., Janocko N.J., Groover O., Loring D.W., Drane D.L., Karakis I. (2022). Depression and Anxiety in Adult Persons With Epilepsy and Their Caregivers: A Survey-Based Study at a Tertiary Care Center. J. Nerv. Ment. Dis..

[26] Micoulaud-Franchi J.A., Bartolomei F., Duncan R., McGonigal A. (2017). Evaluating quality of life in epilepsy: The role of screening for adverse drug effects, depression, and anxiety. Epilepsy Behav..

[27] Siarava E., Hyphantis T., Katsanos A.H., Pelidou S.H., Kyritsis A.P., Markoula S. (2019). Depression and quality of life in patients with epilepsy in Northwest Greece. Seizure.

[28] Szaflarski J.P., Szaflarski M. (2004). Seizure disorders, depression, and health-related quality of life. Epilepsy Behav..

[29] Zigmond A.S., Snaith R.P. (1983). The hospital anxiety and depression scale. Acta Psychiatr. Scand..

[30] Malasi T.H., Mirza I.A., el-Islam M.F. (1991). Validation of the Hospital Anxiety and Depression Scale in Arab patients. Acta Psychiatr. Scand..

[31] el-Rufaie O.E., Absood G.H. (1995). Retesting the validity of the Arabic version of the Hospital Anxiety and Depression (HAD) scale in primary health care. Soc. Psychiatry Psychiatr. Epidemiol..

[32] ePROVIDE-Mapi Research Trust [Internet] Official HADS|Hospital Anxiety Depression Scale Distributed by Mapi Research Trust|e.P.R.OVIDE. https://eprovide.mapi-trust.org/instruments/hospital-anxiety-and-depression-scale.

[33] Cramer J.A., Perrine K., Devinsky O., Bryant-Comstock L., Meador K., Hermann B. (1998). Development and cross-cultural translations of a 31-item quality of life in epilepsy inventory. Epilepsia.

[34] Merrouni M.A., Idrissi A.J., Lamkaddem A., Kava A.C.F., El Fakir S., Souirti Z. (2021). Moroccan Arabic version of the Quality of Life Inventory in Epilepsy (QOLIE-31): Translation, cultural adaptation and psychometric validation. East. Mediterr. Health J..

[35] Quality of Life in Epilepsy Inventory (QOLIE-89 and QOLIE-31) [Internet]. https://www.rand.org/health-care/surveys_tools/qolie.html.

[36] Phabphal K., Sattawatcharawanich S., Sathirapunya P., Limapichart K. (2007). Anxiety and depression in Thai epileptic patients. J. Med. Assoc. Thai..

[37] Stefanello S., Marín-Léon L., Fernandes P.T., Li L.M., Botega N.J. (2011). Depression and anxiety in a community sample with epilepsy in Brazil. Arq. Neuropsiquiatr..

[38] Addis B., Wolde M., Minyihun A., Aschalew A.Y. (2021). Prevalence of depression and associated factors among patients with epilepsy at the University of Gondar Comprehensive Specialized Hospital, Northwest Ethiopia, 2019. PLoS ONE.

[39] Altwijri R.M., Aljohani M.S., Alshammari H.K. (2021). Quality of life among epileptic patients in Qassim Region, KSA. Neurosciences (Riyadh).

[40] Caetano R., Vaeth P.A., Mills B., Canino G. (2016). Employment Status, Depression, Drinking, and Alcohol Use Disorder in Puerto Rico. Alcohol. Clin. Exp. Res..

[41] Aryal S., Jha A. (2020). Quality Of Life in Epilepsy Patients: A cross sectional study. J. Psychiatr. Assoc. Nepal.

[42] Winefield A.H., Tiggemann M., Winefield H.R. (1991). The psychological impact of unemployment and unsatisfactory employment in young men and women: Longitudinal and cross-sectional data. Br. J. Psychol..

[43] Velissaris S.L., Saling M.M., Newton M.R., Berkovic S.F., Wilson S.J. (2012). Psychological trajectories in the year after a newly diagnosed seizure. Epilepsia.

[44] Kimiskidis V.K., Triantafyllou N.I., Kararizou E., Gatzonis S.S., Fountoulakis K.N., Siatouni A., Loucaidis P., Pseftogianni D., Vlaikidis N., Kaprinis G.S. (2007). Depression and anxiety in epilepsy: The association with demographic and seizure-related variables. Ann. Gen. Psychiatry.

[45] Li P., Lin J., Wu C., Huang S., Zhu S. (2021). The impact of social factors, especially psychological worries on anxiety and depression in patients with epilepsy. Epilepsy Behav..

[46] Vitturi B.K., Mitre L.P., Kim A.I.H., Gagliardi R.J. (2021). Prevalence and Predictors of Fatigue and Neuropsychiatric Symptoms in Patients with Minor Ischemic Stroke. J. Stroke Cerebrovasc. Dis..

[47] Operto F.F., Verrotti A., Marrelli A., Ciuffini R., Coppola G., Pastorino G.M.G., Striano P., Sole M., Zucca C., Manfredi V. (2020). Cognitive, adaptive, and behavioral effects of adjunctive rufinamide in Lennox-Gastaut syndrome: A prospective observational clinical study. Epilepsy Behav..

